# Evolution of the Phase Composition in a Nickel-Predominant NiTi Shape Memory Alloy During High-Energy Ball Milling

**DOI:** 10.3390/ma18081882

**Published:** 2025-04-21

**Authors:** Tomasz Goryczka, Grzegorz Dercz, Maciej Zubko

**Affiliations:** Institute of Materials Engineering, University of Silesia in Katowice, 75 Pułku Piechoty 1A, 41-500 Chorzów, Poland; grzegorz.dercz@us.edu.pl (G.D.); maciej.zubko@us.edu.pl (M.Z.)

**Keywords:** NiTi alloys, high-energy ball milling, XRD, SEM, TEM

## Abstract

Three alloys differing in their nominal chemical composition (Ni_50_Ti_50_, Ni_51_Ti_49_, and Ni_52_Ti_48_) were produced in the form of powders using high-energy ball milling. Their microstructure, morphology, structure, and phase composition were studied using the X-ray diffraction technique, scanning, and transmission electron microscopy. For the detailed structural analysis, the Rietveld method was used. The results show that each of the alloys consists of three fractions: fine, medium, and thick. The fractions varied in particle/agglomerate size from 200 nm to 800 μm. Additionally, they varied in phase composition. The fine fraction comprised a mixture of amorphous and nanocrystalline phases. Additionally, the medium and coarse phases showed crystalline solid solutions formed on the bases of nickel or titanium, as well as a crystalline *bcc* phase—a precursor of the parent phase (B2). The largest contribution in the alloy powders, over 80%, comes from the amorphous–nanocrystalline mixture (ANM). The increase in the nickel content resulted in an increase in ANM quantity of 3 wt.%. Similarly, the weight content of the titanium-based solid solution increased to about 7 wt.%. In contrast, the quantity of the nickel-based solid solution decreased from 3 wt.% to approximately 1 wt.% in the Ni_50_Ti_50_ and Ni_52_Ti_48_ alloys.

## 1. Introduction

Binary NiTi shape memory alloys (SMAs), with a chemical composition close to an equiatomic one, show the shape memory effect (SME), which is related to the reversible martensitic transformation (MT). These effects occur only in the intermetallic β-phase (the *bcc* type of crystal lattice) containing from 49.5 at.% to about 54 at.% nickel. The temperature range of its occurrence in the phase equilibrium system is also limited. It is assumed to be stable from about 630 °C to about 1300 °C. The neighbor phases in the phase equilibrium system are the Ni_3_Ti, formed at a higher nickel content, and the Ti_2_Ni, which occurs at a higher titanium content [1]. At room temperature, the β-phase is metastable. It loses its stability in alloys containing nickel predominance when heated at temperatures above 300 °C [2]. Because of the β-phase decay, equilibrium (Ni_3_Ti, Ti_2_Ni) and non-equilibrium phases (e.g., Ni_4_Ti_3_) can be formed, which do not participate in the martensitic transformation and do not contribute to the shape memory effects [1,3].

The MT and SME are sensitive to changes in the chemical composition of the alloy. Primarily, the nature and course of the martensitic transformation depend on the nickel content. Modification of the chemical composition influences the temperature range of its occurrence, as well as its single- or multi-step course [4]. It is assumed that, as the titanium content increases, the characteristic transformation temperatures also increase. An increase in titanium content of 0.2 at.% causes an increase in transformation temperatures of approximately 30 °C [5]. Changing the nickel content in the alloy has the opposite effect. Increasing the nickel content by 0.1 at.% causes the transformation temperatures to decrease by 20 °C [6]. Alloys containing 50 at.% nickel and 50 at.% titanium reveal a single-step B2↔B19′ transformation, while alloys with higher nickel content show a two-step transformation with the sequence B2↔R↔B19′ [7]. Consequently, selecting the alloy’s chemical composition allows the temperature range and the degree of martensitic transformation to be controlled for specific practical applications.

Not only does the chemical composition of the alloy affect the course of the martensitic transformation, but structural changes introduced by technology and the method of producing and processing play a key role. Most production methods make it possible to obtain them in bulk, ribbons, or strips [8]. In order to be applied as a composite material component, a powder of micrometric size is expected. There are known methods for obtaining titanium alloys in the form of powders with a micrometric grain size using the phenomenon of atomization (gas, plasma, water) or powder metallurgy [9,10,11]. However, due to the specificity of NiTi alloys, not all of them are suitable for their production. The most common are powder metallurgy methods—including high-energy ball milling (HEBM)—and developing methods based on the phenomenon of gas atomization (GA). In the case of NiTi alloys, the published literature data mainly concern those with a chemical composition close to equiatomic [12,13]. Technology using GA methods has several limitations that can affect the properties of the NiTi alloy. One of them involves impurities picked up from the crucible by the powders, affecting the chemical composition, agglomeration of particles, or the impact of oxygen pollution [14]. Therefore, based on experience in producing NiTi alloy powders via high-energy milling, the impact of some unfavorable technological factors occurring in GA during the chemical and phase composition can be reduced or eliminated using HEBM. First, the powders are not contaminated with elements from the container material and/or milling balls. In addition, by using lower speeds at the expense of extending the time, it is possible to avoid an increase in the temperature of the charge and, thus, the unfavorable phenomenon of the oxidation of titanium.

These features, produced by the technology of high-energy ball milling, formed the basis of this study, which aimed to produce a NiTi alloy with a diverse chemical composition and to characterize the produced alloys. Because the technology based on high-energy milling allows alloys to be obtained in the form of powders, we attempted to do so for NiTi alloys characterized by the shape memory effect. In this study, HEBM was used to produce an alloy with the nickel content increased, at the cost of titanium, by 1% and 2%. The effect of the nickel content on the evolution of the phases formed during milling was studied and compared to the alloy with an equiatomic chemical composition.

## 2. Materials and Methods

Alloys with the nominal chemical composition Ni(_50+x_)Ti(_50−x_) (where x = 0; 1 or 2) were produced using high-energy ball milling with a batch mass of 20 g. Commercial powders of elements with a purity of 99.7% were used (provided by KAMB Import-Export, Warsaw, Poland). As was suitable for the nominal chemical composition, the weights were milled in a high-energy Fritsch Pulverisette 7 premium line planetary ball mill (FRITSCH GmbH—Milling and Sizing, Idar-Oberstein, Germany). Steel balls with a diameter of 10 mm were used, and their total weight-to-batch mass was 10:1. Milling was carried out in an 80 mL stainless steel container in an argon protective atmosphere for 140 h at a speed of 250 rpm [15].

Microstructure observations were carried out using a scanning electron microscope (SEM) JEOL JSM-6480 (JEOL USA, INC., Peabody, MA, USA) with an energy-dispersive X-ray spectroscopy detector (EDS) (Bruker, Billerica, MA, USA). The powders of alloying elements and alloys were deposited on a conductive carbon tape. Observations at higher magnifications were carried out using a transmission electron microscope (TEM) JEM JEOL 3010 (JEOL USA, INC., Peabody, MA, USA), operating at 300 kV and equipped with a Gatan 2 k × 2 k Orius™ 833SC200D CCD camera (Gatan Inc., Pleasanton, CA, USA). Details of the sample preparation for the TEM studies are described in [16]. Based on microscopic images, the average grain size was determined. The grain size range, obtained from the measurements, was divided into 8 to 10 clusters. The average grain size value and standard deviation were determined from refinement using the logarithmic normal function [17]. Calculations were carried out using OriginPro software (version 2024b) from OriginLab.

The analysis of the phase composition of the alloying elements, as well as the produced alloys, was carried out based on measured X-ray diffractograms. The measurements were completed using an X-ray diffractometer X’Pert-PRO (Malvern Panalytical Ltd., Malvern, UK) equipped with an X-ray tube with Cu_Kα1and2_ radiation (λ_Kα1_ = 1.5406 Å and λ_Kα2_ = 1.5445 Å). The diffractograms were measured in the 2θ angular range from 20° to 140° using the step-scan method, with the counting time adjusted to obtain high-quality diffractograms. Phase identification was performed using the International Center for Diffraction Data (ICDD) PDF-4 database. Crystallographic data from the identified phases were the basis for developing a model of unit cells in the Rietveld analysis. As a least-squares method, the Rietveld refinement was used to refine the details of the structure; on this basis, the theoretical X-ray diffraction pattern was calculated and compared with one obtained from the measurements (experimental). In the case of multiphase materials, in addition to crystallographic data, this method allows the phase’s quantity to be determined [18]. Calculations were performed using the LHPM (Rietica) computer program—version 4.2 [19]. Offset, or the shift in the detector’s zero, was verified based on measurements made for the LaB_6_ standard [20]. Fitting was carried out using the pseudo-Voight diffraction profile line. A fifth-degree polynomial was used for background determination. In addition, the sample displacement parameter was also included in the global parameters. Parameters such as unit cell dimensions, atomic coordinates, site occupancy parameters, the peak half-width parameters of U, V, and W, and a peak shape parameter (Gamm0) were individually adjusted for each phase. The calculations were carried out following the commonly used guidelines [21]. They were stopped when the fitted variable parameter did not cause a change in the reliability of the main factors’ R by 0.1 [22].

## 3. Results and Discussion

### 3.1. Characterization of Elemental Powders

**Figure 1:**
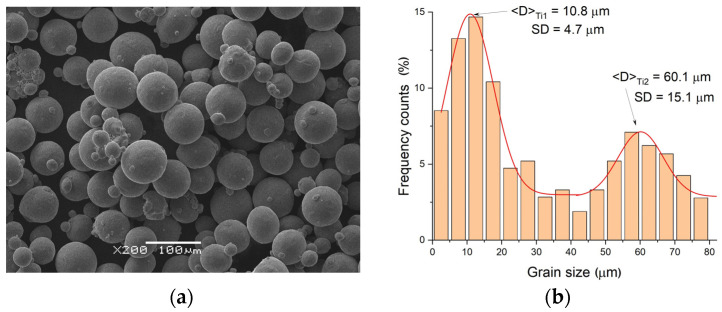
SEM image of titanium powder (**a**) and distribution of the grain size (**b**).

**Figure 2:**
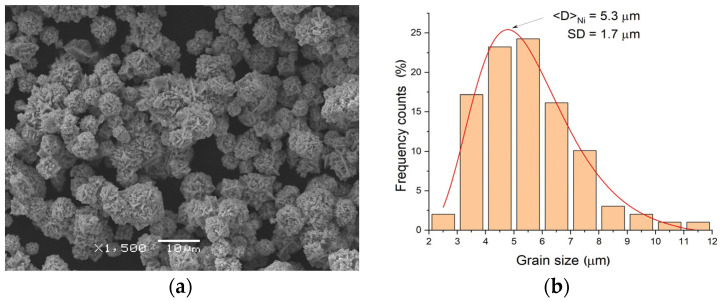
SEM image of nickel powder (**a**) and distribution of the grain size (**b**).

Before alloy production, powders of the alloying elements were characterized in terms of their morphology and average grain size (Figure 1). Grains formed in a spherical shape (Figure 1a) characterized the titanium powder. Two fractions were clearly distinguishable: fine, with an average size of about 11 μm, and large, with an average size of 60 μm (Figure 1b). Unlike titanium, the nickel powder exhibited better homogeneity in terms of the grain size. The grains were in a regular shape, but their surfaces were covered with fine plates (Figure 2a). Microscopic observations confirmed the presence of only one fraction with an average grain size of 5.3 μm (Figure 2b).

**Figure 3:**
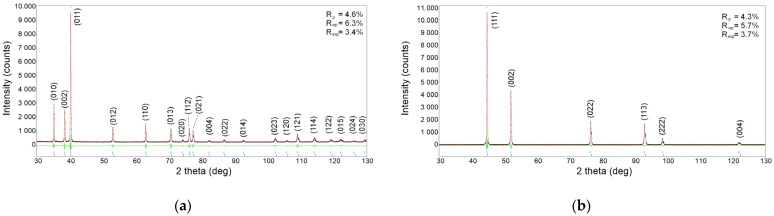
Results of the Rietveld refinement carried out for titanium (**a**) and nickel (**b**) powders. Red line denotes calculated diffraction pattern, black dots—measured one. The green line shows difference between calculations and measurement.

In order to confirm the phase composition of the alloying element, diffractograms were measured, and a Rietveld analysis was performed [21]. For the crystallographic unit cell model, titanium and nickel lattice parameters were obtained from the International Center for Diffraction Data (ICDD) files: PDF-4 no. 00-044-1294 and 00-087-0712 for Ti and Ni, respectively. The graphical results of the refinement of the calculated diffractogram to the experimental ones are presented in Figure 3. In addition, the figure contains the values of factors (R_p_, R_wp_ and R_exp_) characterizing the quality of the fitting referring to the peaks (R_p_), the entire diffractogram (R_wp_), and the quality of the measurement (R_exp_) [22]. The R_exp_ factor reflects the quality of the experiment performed and depends on the measurement range, the recording step, the counting time, and the count statistics. Diffractograms with R_exp_ values of 3 to 5% are considered to be of high quality and constitute the basis for the quantitative calculations of phases with weight fractions below 1%. In the case of the studied powders, the values of the coefficients characterizing the fit and measurement meet the above requirements. Based on the performed calculations, the lattice parameters were determined. The results are presented in Table 1. Their values were comparable to the pattern data from the ICDD files. Moreover, no additional phases or impurities were found for either of the alloying elements. This confirms the high quality of the alloying powders.

### 3.2. Characterization of As-Milled Alloys

In the first hours of grinding, the powders of the alloying elements formed larger agglomerates composed of the same or both alloying elements. Gradually extending the milling time to 40 h caused an increase in the agglomerate’s average diameter to approximately 200 μm. As the process progressed, milling led to reactions between the agglomerates themselves and between the agglomerates and the balls, as well as the walls of the milling container. As a result, some of agglomerates were fragmented and their average size decreased. In contrast, some of them incorporated finer grains and continuously increased their average diameter. Due to the evolution of the powder morphology, after approximately 60 h, two fractions differing in diameter began to form. Extending the milling process to 140 h led to the formation of three distinguishable powder fractions [23].

**Figure 4:**
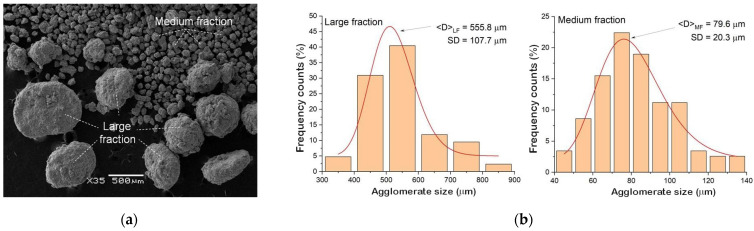
SEM image of the Ni_51_Ti_49_ powder mixed for 140 h (**a**) and plots of agglomerates’ size distribution determined for the large and medium fractions (**b**).

**Figure 5:**
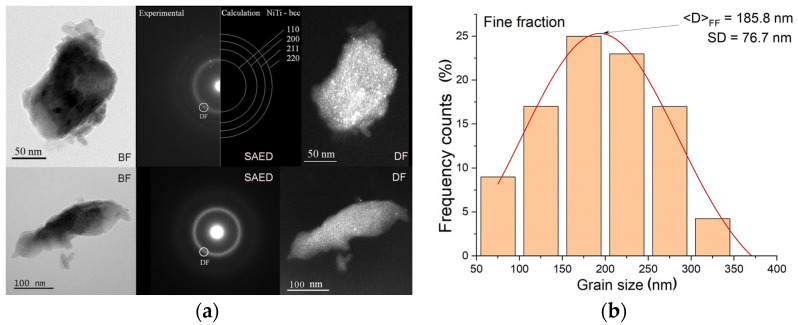
Examples of TEM images: BF, SAED, and DF (**a**) for the fine fraction of the Ni_51_Ti_49_ alloy mixed for 140 h and the plot of grain size distribution (**b**).

This behavior was observed for each of the produced alloys. Regardless of their chemical composition, the alloys consisted of three fractions: fine, medium, and coarse. Figure 4a shows an example of the SEM image observed for the Ni_51_Ti_49_ alloy. Powders of the other two alloys revealed similar morphologies. The medium fraction had the most significant weight content—about 85%. The amount of coarse and fine fractions amounted to several percent each. The coarse fraction consisted of agglomerates ranging in size from approximately 300 μm to 800 μm. The average agglomerate size was 556 μm (Figure 4b). These agglomerates were mainly spherically shaped. However, among them were discs with a thickness of about 200 μm. This occurred due to plastic deformation resulting from the collision of agglomerates with the balls and with the container’s walls. In turn, the medium fraction consisted only of agglomerates characterized by spherical shapes with an average size of 80 μm. However, this fraction consisted of agglomerates with diameters ranging from about 45 μm to about 135 μm. The finest fraction was formed from grains with an average size of 186 nanometers (Figure 5). In fact, they were the building blocks of agglomerates occurring in the medium and coarse fraction. They consisted of irregularly shaped grains ranging from 75 nm to about 330 nm (Figure 5b). Similar fine grains were observed in the Ni_50_Ti_50_ as well as the Ni_52_Ti_48_ alloy.

The microscopic observations and the diffraction images confirmed that the fine fraction is an amorphous–nanocrystalline mixture. The electron diffraction images showed circles whose radii, converted into interplanar distances, corresponded to the distances characteristic of the disordered *bcc* phase, which is the precursor of the parent phase in NiTi alloys (Figure 5a).

**Figure 6:**
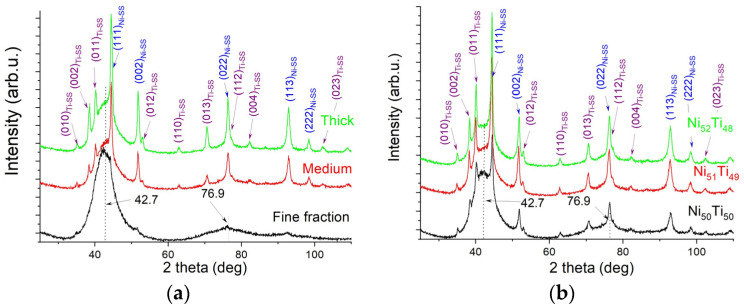
Set of X-ray diffraction patterns measured for fractions in the milled Ni_52_Ti_48_ alloy (**a**) and alloys varying in nickel content; the sample contained a mixture of all three fractions (**b**).

The study of the phase composition of individual fractions was carried out based on measured X-ray diffraction patterns. The results are summarized in Figure 6a. In the diffractogram registered for the fine fraction, there were only two maxima at the 2θ positions of 43° and 77°, characterized by a broadened half-width of peak (FWHM)—about 6°. In X-ray diffraction, this phenomenon is interpreted as coming from coherent scattering only and indicates an amorphous or an ultra-fine nanocrystalline state. The observations, made using the transmission microscope, proved that the fine fraction was an amorphous–nanocrystalline mixture (ANM). This mixture was also present in the medium and coarse fractions, as evidenced by peaks with extended half-widths occurring in the similar position. Additionally, diffraction lines characteristic of crystalline phases appeared in the diffractograms measured for the thick and medium fractions. The identification showed that they belonged to solid solutions based on titanium (Ti-SS) and nickel (Ni-SS): see Figure 6a.

**Figure 7:**
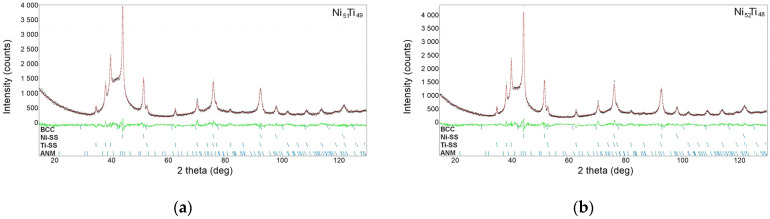
Example of the Rietveld refinement carried out for the Ni_52_Ti_48_ (**a**) and Ni_52_Ti_48_ (**b**) alloys. Red line denotes calculated diffraction pattern, black dots—measured one. The green line shows difference between calculations and measurement.

The result was the phase composition of the produced alloys. Thus, consistently measured X-ray diffractions patterns for the Ni_50_Ti_50_, Ni_51_Ti_49_, and Ni_52_Ti_48_ alloys confirmed the presence of the ANM and two solid solutions: Ti-SS and Ni-SS (Figure 6b). Similarly to the powders of the alloying elements, the measured diffractograms for the produced alloys were fitted using the Rietveld method. As shown in another study [15], the best crystallographic model for describing the arrangement of atoms representing the ANM is the structure of martensite with a monoclinic type of lattice, i.e., B19′. The unit cell parameters for the *bcc* phase were the crystallographic data of the parent phase B2. Examples of the refinement of the alloys Ni_51_Ti_49_ and Ni_52_Ti_48_ are shown in Figure 7. The unit cell parameters determined from the calculations for individual phases are summarized in Table 2. The results of calculation showed that the titanium-based solid solution’s *a*_0_ and *c*_0_ unit cell parameters are lower than those for titanium powder in its initial state (Table 1). The opposite situation was true of the nickel-based solid solution, in which the unit cell parameter *a*_0_ was larger than that determined for nickel powder. Considering that titanium’s atomic radius is larger than nickel’s (Ti—1.4 Å, Ni—1.35 Å) [24], the crystal lattice of a titanium-based solid solution is contracted. Its *a*_0_ parameter decreased by approximately 0.1% and the *c*_0_ parameter by 0.15%. This feature is characteristic of the hexagonal crystallographic system, in which the unit cell deforms more easily along the Z axis than the X or Y one. This confirms the limited solubility of nickel in titanium, which, due to the phase equilibrium system of the binary NiTi alloy, is less than 1% at room temperature [1]. Conversely, the crystal lattice of the nickel-based solid solution underwent an expansion. The *a*_0_ parameter increased by approximately 6%. This indicates the superior solubility of titanium in the nickel crystal lattice. According to the NiTi phase equilibrium system, this solubility can reach 10% [1]. However, it should be remembered that the conditions during the milling process, especially the high-energy ones, are far from an equilibrium state. Hence, high-energy milling makes it possible to extend the solubility ranges of individual components.

The results obtained from the Rietveld refinement were also used to quantify the content of individual phases in the produced alloys (Table 3). In the case of the quantitative phase analysis performed using the Rietveld method, the fitting quality of the calculated and measured diffractograms is essential. Therefore, Table 3 lists the values of the most critical coefficients characterizing the fitting quality: R_p_, R_wp_, R_exp_. The values of these coefficients did not exceed 6.5% and proved the high quality of the refinement as well as the experimental data.

The calculated weight content of the individual phases clearly indicates that the dominant phase is the amorphous–nanocrystalline mixture. Its content is comparable for the Ni_50_Ti_50_ and Ni_51_Ti_49_ alloys and amounts to approximately 81 wt.%. Increasing the nickel content to 52 at.% and simultaneously reducing the titanium content to 48 at.% resulted in an increase in the weight content of this phase to 83 wt.%. Simultaneously, the content of the nickel-based solid solution decreased from 4 wt.% to almost 1 wt.%. This fact proves the enrichment of the amorphous–nanocrystalline mixture in nickel. Consequently, the content of the titanium-based solid solution increased from 0.3 wt.% to almost 7 wt.%. From the point of view of the final product, the alloy’s most crucial components are the disordered *bcc* phase and the ANM. The amount of the *bcc* phase decreased from 16 wt.% to almost 10 wt.% with increased nickel content in the alloy. On the contrary, the amount of ANM increased from of 3 wt.% with the increase in nickel. The results indicate that such an increase favors the formation of the ANM. This suggests that most of the alloy is amorphized or undergoes nanocrystalline fragmentation. Regarding the occurrence of a reversible martensitic transformation, the presence of both phases is beneficial. After the crystallization process, the amorphous phase transforms into a crystalline form with a B2 structure, while the disordered *bcc* phase also takes the form of the B2 phase [23].

An unfavorable phenomenon that occurs during high-energy milling is the deposition of powders on the inner surface of the container, as well as on the surface of the milling balls. This phenomenon leads to a loss of approximately 10% of the charge mass. The layers created on the surface of the balls can be up to about 140–150 μm thick. Sometimes, it detaches during milling, and the detached material returns to the ground powder. In the first stage of grinding, these layers are formed from plastically deformed grains of alloying elements that are also welded into the surface of the balls/container. A positive aspect of these layers forming is the protection they provide against the material from which the balls are made. They prevent the alloy powders from experiencing milling pollution. As the grinding time increases, new, deformed agglomerates/grains join the formed layer surface, causing the layer’s thickness to increase. In this way, the history of developing the chemical composition is created. In order to investigate the changes occurring in the chemical composition during grinding, a cross-section of the ball was made and observed in a scanning electron microscope.

**Figure 8:**
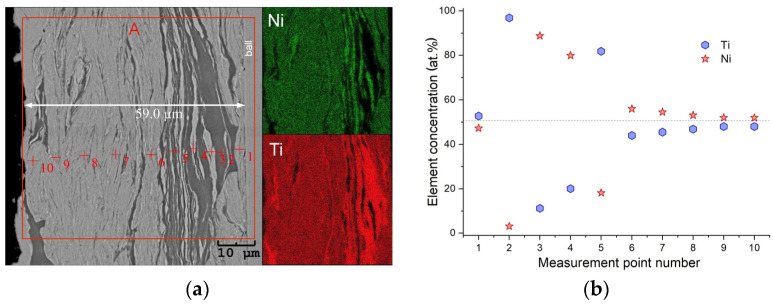
SEM-BSE image of a cross-section of a ball used to mill the Ni_52_Ti_48_ alloy and measured element distribution maps from area “A” (**a**) and chemical composition determined at points 1–10 based on EDS measurements—dashed line represents nominal chemical composition (**b**).

Figure 8a shows an example of an SME-BSE image of the cross-section of the ball used in the Ni_51_Ti_49_ alloy grinding. A layer about 60 μm thick surrounded its surface. The contrast, from observations made using backscattered electrons, produced information about areas composed of elements dominated by titanium (dark areas), nickel (bright areas) and the emerging NiTi alloy (a greyish area). This was confirmed by the measured element distribution maps from the region marked as “A”. They indicated appropriate areas characterized by higher concentrations of alloying elements. Additionally, the measurement of the chemical composition at points marked from 1 to 10 and plotted in Figure 8b revealed the progressive homogeneity of the chemical composition as the grinding time increased. The results proved that the areas of the layer formed near the sphere’s surface were formed from solid solutions based on nickel and titanium. This fact is confirmed by the measurement results at points 2 to 5. Further increases in the milling time caused progress in the diffusion of the elements and led to the homogenization of the chemical composition (measurement points: 6 to 10). At point no. 10, the titanium content was 48.2 at.%, and the nickel content was 51.8 at.%. These values were close to the nominal composition. A similar trend occurred in the case of the other two alloys.

**Figure 9:**
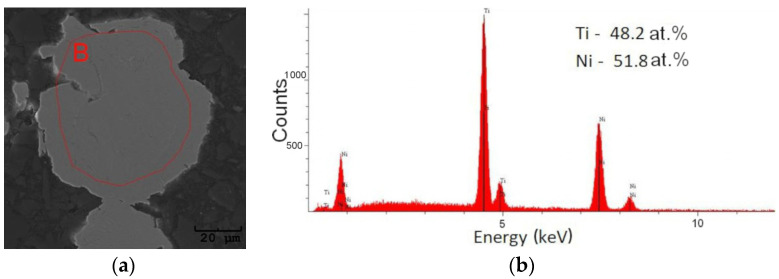
Example of SEM image of the powder’s cross-section for the Ni_52_Ti_48_ alloy (**a**) and the EDS spectrum measured as from area “B” (**b**).

The EDS measurement conducted for the near-surface areas of the layer reflects the chemical composition of the produced alloy’s powders. To confirm these findings, EDS measurements were carried out on the cross-sections of the powders, and the average chemical composition was determined. Measurements were performed on dozens of grains’ cross-sections in all of the produced alloys. An example of such a measurement carried out for the Ni_52_Ti_48_ alloy is shown in Figure 9. The average chemical composition is given in Table 4.

**Figure 10:**
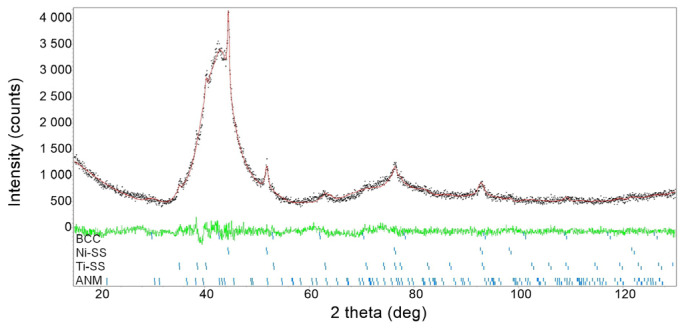
Results of the Rietveld refinement carried out for materials collected from the milling balls and the container used for the Ni_51_Ti_49_ alloy. Red line denotes calculated diffraction pattern, black dots—measured one. The green line shows difference between calculations and measurement.

The values of the standard deviations indicate discrepancies in the nickel and titanium content in individual grains. Comparing the obtained values to the nominal composition shows a slight predominance of titanium content. These differences range from 0.1 to 0.2 at.%. This effect may be related to the deposition of powders of the alloying elements on the surface of the container and balls during the first grinding stages. In order to study this phenomenon, for the Ni_51_Ti_49_ alloy, scratched powder fragments were collected from the surface of the container and milling balls. For this material, an X-ray diffraction pattern was measured and fitted using the Rietveld method (Figure 10). First, the phase identification showed that this material consists of four previously identified phases for alloy powder. The calculations carried out using the Rietveld method; the results revealed that the amount of the AMN phase was 91.7 wt.%, the *bcc* phase was 6.1 wt.%, Ni-SS was 1.3 wt.%, and Ti-SS was 0.9 wt.%. This means that the slight differences between the nominal chemical composition and the measured ones result from a slight nickel loss.

## 4. Conclusions

The results obtained from studies carried out on NiTi alloys produced via high-energy milling are summarized in the following conclusions:

1. High-energy milling, carried out for 140 h, produced NiTi alloys with a chemical composition close to the nominal one, in the form of powders consisting of three fractions differing in the average grain/agglomerate size: 200 nm; 80 μm; and 556 μm.
2. The alloys consisted of four basic phases: amorphous–nanocrystalline mixtures, solid solutions based on nickel and titanium, and a phase with a *bcc* structure, a precursor of the parent phase undergoing a reverse martensitic transformation.
3. Regardless of the chemical composition of the produced alloy, the unit cell of the titanium-based solid solution underwent contraction; the lattice parameter decreased by approximately 0.15%, indicating a limitation in the content of dissolved nickel. In turn, the nickel unit cell parameters expanded, revealing the possibility of dissolving approximately 6% of titanium.
4. The increase in the nickel content in the NiTi alloys contributed to an increase in the weight content of the amorphous–nanocrystalline mixture. The content of this mixture in the produced alloys was over 80 wt.%.

## Figures and Tables

**Table 1:** Determined lattice parameters and those from the ICDD for powders in their initial states.

Lattice Parameter [Å]				
	*a*_0_		*c*_0_	
	Calculated	ICDD	Calculated	ICDD
Ti	2.951(4)	2.9505 ^1^	4.689(9)	4.6826 ^1^
Ni	3.324(8)	3.5238 ^2^	-	-

^1^ ICDD card no. 00-044-1294; ^2^ ICDD card no. 00-087-0712.

**Table 2:** Determined lattice parameters and those received from the ICDD files for the phase identified in the produced alloys.

Sample	Lattice Parameter [Å]				
	Ti-SS		Ni-SS	*bcc*	
	Calculated		Calculated	Calculated	ICDD ^1^
	*a*_0_	*c*_0_	*a*_0_	*a*_0_	*a*_0_
Ni_50_Ti_50_	2.948(5)	4.683(3)	3.527(6)	3.008(5)	3.015
Ni_51_Ti_49_	2.949(6)	4.682(3)	3.526(1)	3.002(1)	
Ni_52_Ti_48_	2.949(3)	4.683(1)	3.525(9)	3.004(9)	

^1^ ICDD card no. 00-044-1294.

**Table 3:** Phase composition, its weight percentage (dependent on nickel content) and reliability factors of XRD-pattern refinement.

Alloy	Weight Percentage of Phases [%]					Reliability Factors [%]		
	ANM	Solid Solutions		*bcc*	Total	R_p_	R_wp_	R_exp_
		Ni-SS	Ti-SS					
Ni_50_Ti_50_	80.3	3.6	0.3	15.8	100	4.41	5.62	4.10
Ni_51_Ti_49_	80.6	0.9	5.9	12.6	100	4.76	6.20	4.36
Ni_52_Ti_48_	83.0	0.8	6.6	9.6	100	5.06	6.49	4.47

**Table 4:** Nominal and average chemical composition determined for all produced alloys.

Alloy	Content of Alloying Elements [at.%]			
	Nominal		Experimental	
	Ni	Ti	Ni	Ti
Ni_50_Ti_50_	50	50	49.8 ± 0.3	50.2 ± 0.3
Ni_51_Ti_49_	51	49	50.9 ± 0.3	49.1 ± 0.3
Ni_52_Ti_48_	52	48	51.7 ± 0.3	48.3 ± 0.3

## Data Availability

The datasets presented in this article are not readily available due to the ongoing research process, which requires systematic analysis, interpretation, and publication of the results in accordance with the principles of scientific integrity. Requests to access the datasets should be directed to the corresponding author.
